# Polyetheretherketone (PEEK) rods versus titanium rods for posterior lumbar fusion surgery: a systematic review and meta-analysis

**DOI:** 10.1186/s13018-023-03817-2

**Published:** 2023-05-11

**Authors:** Wenhao Li, He Zhao, Chuanhong Li, Tao Liu, Jianbin Guan, Yongdong Yang, Xing Yu

**Affiliations:** 1 Beijing University of Chinese Medicine, Beijing, 100700 China; 2 Dongzhimen Hospital Affiliated to Beijing University of Chinese Medicine, Beijing, 100700 China

**Keywords:** Lumbar fusion surgery, Meta-analysis, PEEK rod, Titanium rod

## Abstract

**Background:**

Rigid fixation, represented by titanium rods, is a widely used fixation technique for lumbar fusion. However, this technique carries the risk of degeneration of adjacent segments. In recent years, the semi-rigid fixation technique represented by PEEK rods has gradually matured, and its effectiveness has been verified by numerous studies. The aim of this study was to systematically evaluate the effectiveness of these two fixation modalities in posterior lumbar fusion surgery.

**Methods:**

Studies meeting the inclusion criteria were searched in PubMed, Cochrane Library, ScienceDirect, Embase, CNKI, and Wanfang databases. After data extraction and quality assessment of included studies, meta-analysis was performed using STATA 15.1 software. The protocol for this systematic review was registered on INPLASY (2021110049) and is available in full on the inplasy.com (https://inplasy.com/inplasy-2021-11-0049/).

**Results:**

Fifteen relevant studies were finally included, including eight prospective studies and seven retrospective studies. The results of meta-analysis showed that in ODI (*P* = 0.000), JOA score (*P* = 0.017), VAS score for lower limb pain (*P* = 0.027), fusion rate of bone graft at week 12 (*P* = 0.001), fusion rate of bone graft at last follow-up (*P* = 0.028), there was a statistical difference between the two groups. The PEEK rod group was superior to the titanium rod group in the above aspects. While in VAS score for LBP (*P* = 0.396), there was no statistical difference between the two groups.

**Conclusion:**

Both PEEK rods and titanium rods are effective fixation materials in lumbar fusion surgery. PEEK rods may be superior to titanium rods in improving postoperative function and improving bone graft fusion rates. However, given the limitations of this study, whether these conclusions are applicable needs further research.

## Background

Lumbar fusion is a commonly used surgical procedure for the treatment of degenerative lumbar spine diseases. And rigid fixation represented by titanium rods is a widely used fixation method in lumbar fusion surgery. It can provide strong stability to the lumbar spine and facilitate implant fusion. However, rigid fixation can significantly alter the distribution of lumbar spine loading, leading to adjacent segment degeneration (ASD) [1–3].

To address this problem, semi-rigid fixation systems represented by polyetheretherketone (PEEK) rods, an inert, semi-crystalline thermoplastic polymer with biocompatible properties [4], have been clinically applied with a stable chemical structure, minimal toxicity, and good mechanical properties such as high strength, good wear resistance, and fatigue properties [5–7]. PEEK materials were first used in spinal surgery in the form of intervertebral fusion devices [8, 9], and since 2007, PEEK rods have been used for dynamic stabilization [5, 10]. After more than a decade of development and improvement, the effectiveness and safety of PEEK rods have been validated by several biomechanical and clinical studies.

Despite these advantages, there are still few studies that systematically compare the effectiveness of PEEK rods and titanium rods in lumbar fusion surgery. Although Selim et al. [11] performed a systematic review in 2018, they included only five studies, four of which were retrospective, and the studies were conducted before 2016 with a small sample size (177 patients, 156 in the PEEK group and 21 in the titanium group). Therefore, we performed a larger sample size meta-analysis with the aim of comprehensively evaluating the effectiveness of PEEK rods versus titanium rods in posterior lumbar fusion surgery to provide stronger evidence to support clinical decision making.

## Methods

This meta-analysis was performed according to the Cochrane Handbook [12], and as it is a systematic review of published studies, ethical approval is not required.

### Inclusion and exclusion criteria

Studies that met the following inclusion criteria were included: (1) Published clinical prospective or retrospective controlled studies. (2) The subjects were patients who received lumbar fusion due to lumbar degenerative diseases. The age, gender and nationality of the patients were not limited. (3) PEEK rod-pedicle screw internal fixation-intervertebral bone graft fusion was used as an intervention measure, and titanium rod-pedicle screw internal fixation-intervertebral bone graft fusion was used as a control measure.

The studies were excluded according to the following criteria: (1) The subjects were combined with lumbar spine trauma, fracture, tumor, infection or combined with coagulation dysfunction, thrombosis, mental system diseases, etc. (2) The research subjects had received lumbar spine surgery. (3) Biomechanics and animal studies. (4) Non-fusion surgery, or a combination of fusion and non-fusion surgery.

### Search strategies

After determining the inclusion and exclusion criteria for this study, two researchers independently searched multiple databases, including PubMed (1966–May 1, 2022), Cochrane Library (1966–May 1, 2022), ScienceDirect (1980–May 1, 2022), Embase (1980–May 1, 2022), China National Knowledge Infrastructure (CNKI) (1980–May 1, 2022) and Wanfang Database (1980–May 1, 2022).

We used the following search terms: polyetheretherketone rod, PEEK rod, Semi-rigid fixation, radiolucent spinal implant, and used the Boolean operators AND or OR. The retrieved studies were gradually screened by two researchers based on title, abstract and full text. After identifying included articles, we traced their references to identify potential articles.

### Data extraction

After the initial screening and secondary screening of the literatures in strict compliance with the established inclusion and exclusion criteria, two independent researchers extracted data from the literatures that met the requirements, and sent them to a third researcher for inspection and verification after the extraction was completed. Any disagreement about the included studies was reached through discussion among all investigators. The main data extracted in this study included: first author's name, year of publication, sample size, patient sex ratio, mean age, intervention method, country, study type, follow-up time, and clinical outcomes.

### Quality assessment

In this study, the Cochrane Risk of Bias Tool [13] was used to evaluate the quality of prospective studies, which included seven assessments: random sequence generation, allocation concealment, blinding of performers and outcome assessors, data integrity, whether there is selective reporting and other aspects of bias. The risk of bias for each aspect was judged as low risk, high risk, or unknown risk, indicated by symbols with different colors. This work was done by two researchers using Review Manager software (RevMan 5.3).

For included retrospective controlled studies, we used the MINORS scale for quality assessment. The scale has 12 items in total, with 0–2 points for each item, with a total score of 24 points. 0 means not reported; 1 means reported but insufficient information; 2 means reported and provided sufficient information.

### Data analysis

We performed statistical analysis using STATA software (version 15.1). Continuous variables were reported as mean difference (MD) and 95% confidence interval (CI), while dichotomous variables were reported as odds ratio (OR) and 95% CI. Statistical heterogeneity was judged according to the *I*^2^ statistic. The greater the *I*^2^, the greater the heterogeneity. If there was heterogeneity in this study (*I*^2^ ≥ 50%), a random-effects model was used; otherwise, a fixed-effects model (*I*^2^ < 50%) was used. In this study, differences were considered statistically significant when *P* < 0.05.

## Results

### Search result

**Fig. 1:**
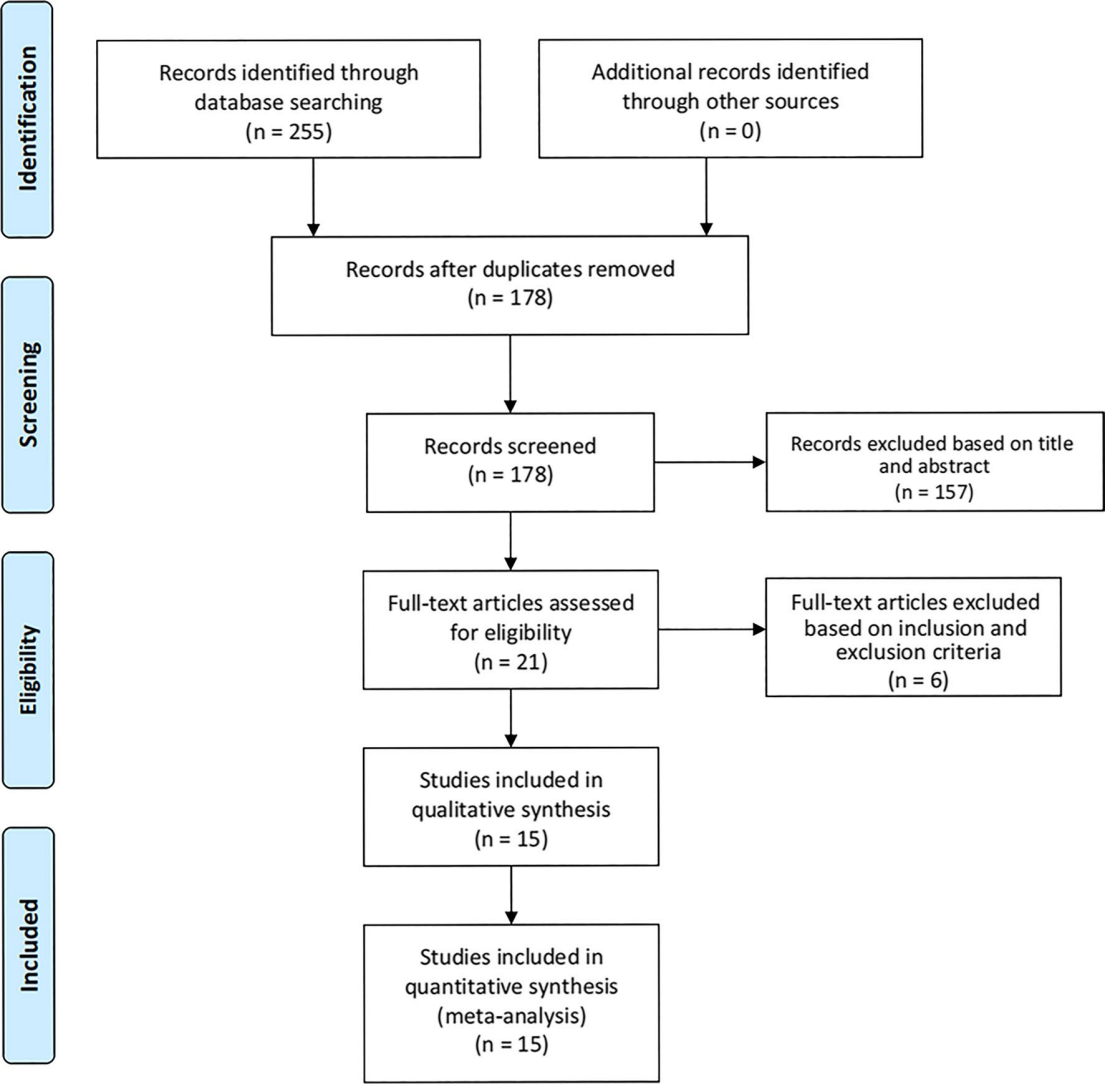
Flow diagram of the study selection process

This meta-analysis has been reported according to the Preferred Reporting Items for Systematic Reviews and Meta-Analysis (PRISMA) Statement [14]. A total of 255 relevant studies were identified from the electronic database, and 178 studies were obtained after deduplication. According to the titles and abstracts of these studies, 21 studies related to this study were further obtained according to the inclusion and exclusion criteria. After careful full-text evaluation of these studies, 15 studies [15–29] were finally included in the final comprehensive analysis. The literatures screening flowchart is shown in Fig. 1, and the basic characteristics of the included studies are shown in Table 1.

**Table 1:** Basic characteristics of the studies include

Authors	Sample size		Total	Gender (female)		Mean age (years)		Intervention		Country	Study type	Follow-up (postoperative)	Outcome assessment
	T	C		T (%)	C (%)	T	C	T	C				
Zhang et al. 2016 [15]	32	31	63	50	48	56.15 ± 2.15	55.35 ± 3.01	PEEK	Ti	China	Prospective	4th, 12th, 24th weeks	ODI Bone graft fusion rate
Sun et al. 2016 [16]	24	24	48	46	50	61.5 ± 5.3	61.3 ± 2.1	PEEK	Ti	China	Prospective	4th, 12th, 24th, 48th months	VAS JOA Bone graft fusion rate
Wang et al. 2020 [17]	27	27	54	67		54.6 ± 4.3		PEEK	Ti	China	Prospective	12th, 24th, 48th weeks	ODI JOA Bone graft fusion rate Adverse event
Li et al. 2019 [18]	21	26	47	45		54.6 ± 4.3		PEEK	Ti	China	Prospective	4th, 12th, 72th,144th weeks	Operation time Intraoperative blood loss Postoperative drainage Length of stay ODI VAS
Tang et al. 2020 [19]	58	48	106	55	58	57.45 ± 7.37	56.39 ± 7.26	PEEK	Ti	China	Prospective	2th, 12th, 48th weeks	Operation time Intraoperative blood loss Postoperative drainage Length of stay ODI VAS Intervertebral ROM Adverse event
Ding et al. 2020 [20]	35	35	70	57	40	56.16 ± 2.16	55.36 ± 3.02	PEEK	Ti	China	Prospective	4th, 12th, 24th weeks	ODI JOA Intervertebral ROM Bone graft fusion rate Adverse event
Qi et al. 2013 [21]	20	21	41	45	48	48.9(38–63)	50.4(32–74)	PEEK	Ti	China	Prospective	12th, 24th, 48th weeks	VAS for LBP VAS for LP JOA JOA recovery rate DH
Huang et al. 2016 [22]	21	20	41	33	40	65.2 ± 4.1	64.7 ± 5.4	PEEK	Ti	China	Prospective	3th, 6th, 12th months	ODI JOA Bone graft fusion rate
Li et al. 2015 [23]	25	25	50	46		44.54 ± 1.35		PEEK	Ti	China	Retrospective	1th, 6th, 12th months	JOA ODI Bone graft fusion rate DH Intervertebral ROM
Han et al. 2016 [24]	16	16	32	53		61.2 ± 1.3		PEEK	Ti	China	Retrospective	1th, 3th, 6th, 12th months	VAS for LBP VAS for LP JOA
Lu et al. 2017 [25]	24	24	48	46		60.2 ± 1.2		PEEK	Ti	China	Retrospective	3th, 6th, 12th months	VAS for LBP VAS for LP JOA
Pang et al. 2020 [26]	30	30	60	67	60	56 ± 13	53 ± 13	PEEK	Ti	China	Retrospective	3th, 6th, 12th, 24th, 36th months	JOA bone graft fusion rate JOA recovery rate
Yang et al. 2015 [27]	24	27	51	46	56	47.5 ± 5.3	49.9 ± 5.3	PEEK	Ti	China	Retrospective	3th, 6th, 12th, 24th months	VAS for LBP VAS for LP ODI Bone graft fusion rate DH
Yu et al. 2012 [28]	13	15	28	31	60	53.2 ± 11.3	55.3 ± 10.3	PEEK	Ti	China	Retrospective	3th, 6th months	JOA Bone graft fusion rate JOA recovery rate
Xu et al. 2012 [29]	20	20	40	45	60	50.35 ± 9.16	56.74 ± 13.86	PEEK	Ti	China	Retrospective	7–16 months	ODI Bone graft fusion rate

PEEK: PEEK rod-pedicle screw internal fixation-intervertebral bone graft fusion; Ti: titanium rod-pedicle screw internal fixation-intervertebral bone graft fusion; ODI: Oswestry Disability Index; VAS: Visual Analogue Scale score; LBP: low back pain; LP: leg pain; JOA: Japanese Orthopeadics Association score; ROM: range of motion; DH: disc space height

### Quality assessment

**Fig. 2:**
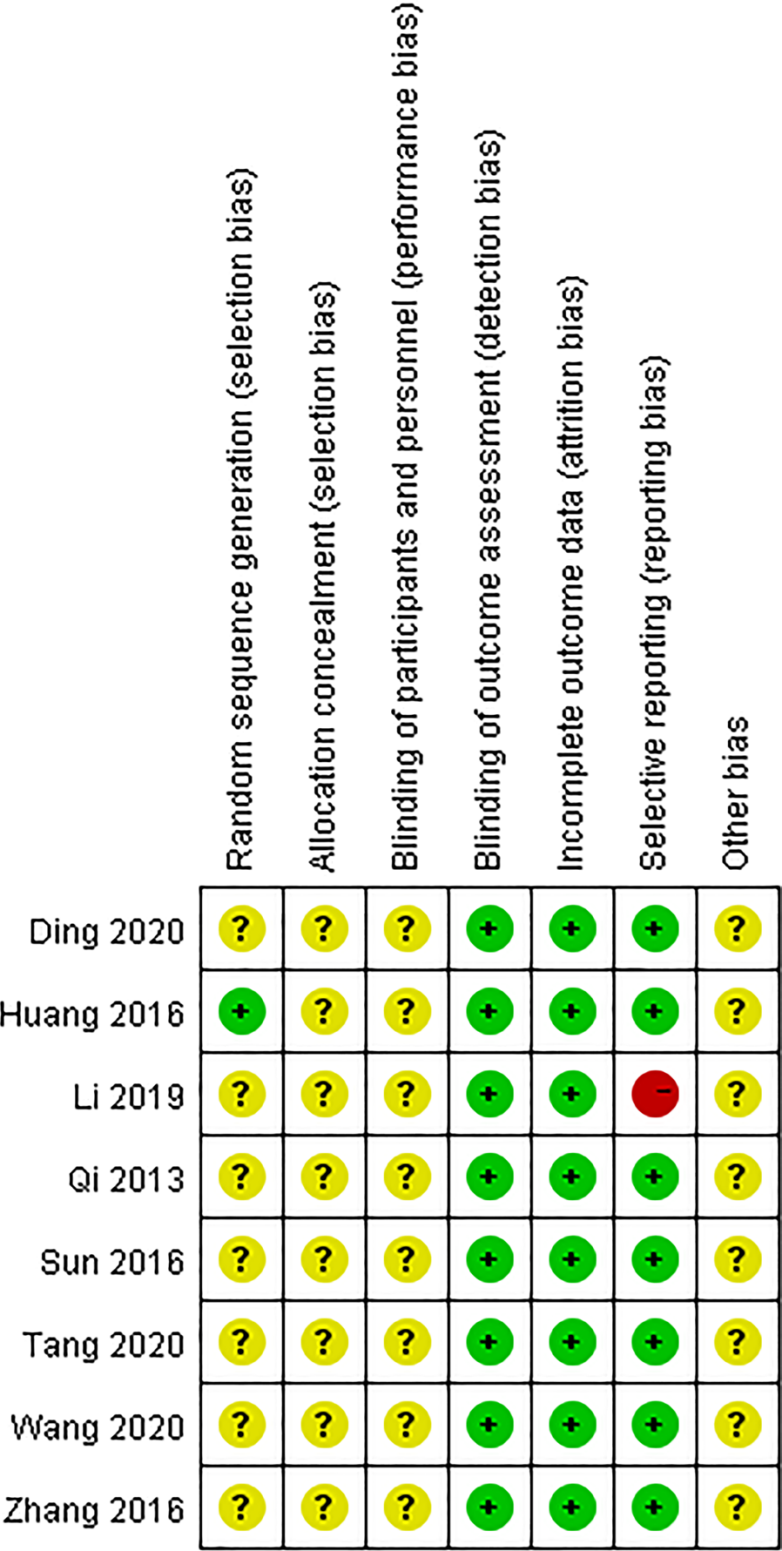
Risk of bias summary: +, low risk of bias; −, high risk of bias; ?, bias unclear

Except for Huang et al. [22] 's study using the random number table for randomization, the remaining 7 prospective studies did not mention randomization, allocation concealment, and blinding, so the risks in these three aspects were unknown. We speculate that this may be related to the particularity of surgical treatment and ethical requirements. Before performing surgery, the patient's right to know must be guaranteed, and the patient's personal wishes must be fully listened to, so it is difficult to implement randomization, allocation concealment, and blinding. In terms of selective reporting, since the study by Li et al. [18] did not report data on the rate of bone graft fusion, it was considered high risk, and the rest of the studies were low risk. No patients were withdrawn or lost to follow-up in all studies, and the data were complete. As shown in Fig. 2.

The seven included retrospective studies did not adequately describe the coherence of included patients, the objectivity of outcome indicators, whether the sample size was estimated, and whether the control group was synchronized. Therefore, 1 point is awarded for each of these aspects. See Table 2 for details.

**Table 2:** Quality assessment of retrospective studies

	Li 2015	Han 2016	Lu 2017	Pang 2020	Yang 2015	Yu 2012	Xu 2012
Purpose of the research is clear	2	2	2	2	2	2	2
Consistency of enrolled patients	1	1	1	1	1	1	1
Collection of expected data	2	2	2	2	2	2	2
Outcomes that appropriately reflect the purpose of the study	2	2	2	2	2	2	2
Objectivity of outcome measures	1	1	1	1	1	1	1
Adequate follow-up time	2	2	2	2	2	1	2
Loss to follow-up rate is less than 5%	2	2	2	2	2	2	2
Estimated sample size	1	1	1	1	1	1	1
Appropriate selection of the control group	2	2	2	2	2	2	2
Control group synchronization	1	1	1	1	1	1	1
Baseline comparable between groups	2	2	2	2	2	2	2
Statistical analysis is appropriate	2	2	2	2	2	2	2
Total	20	20	20	20	20	19	20

### Results of the meta-analysis

#### ODI

**Fig. 3:**
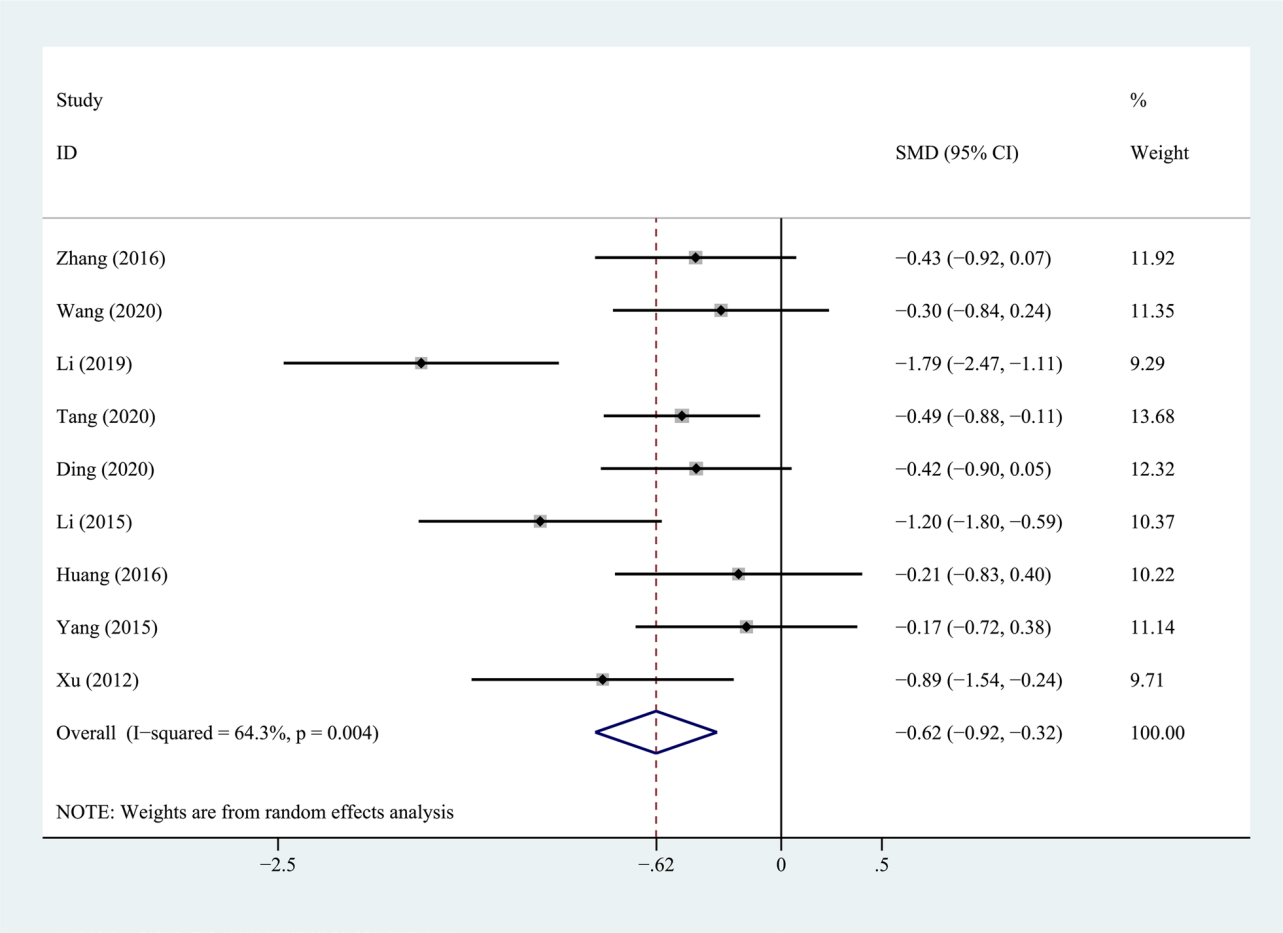
Forest plot of ODI between PEEK and titanium rod

Nine studies compared ODI scores between PEEK rods and titanium rods, as shown in Fig. 3. The heterogeneity test showed that there was significant heterogeneity between studies (*P* = 0.004, *I*^2^ = 64.3%), so a random-effects model was used to analyze the data. The combined results showed that the difference between two groups was statistically significant (*P* = 0.000).The PEEK rod group was superior to the titanium rod group.

#### JOA score

**Fig. 4:**
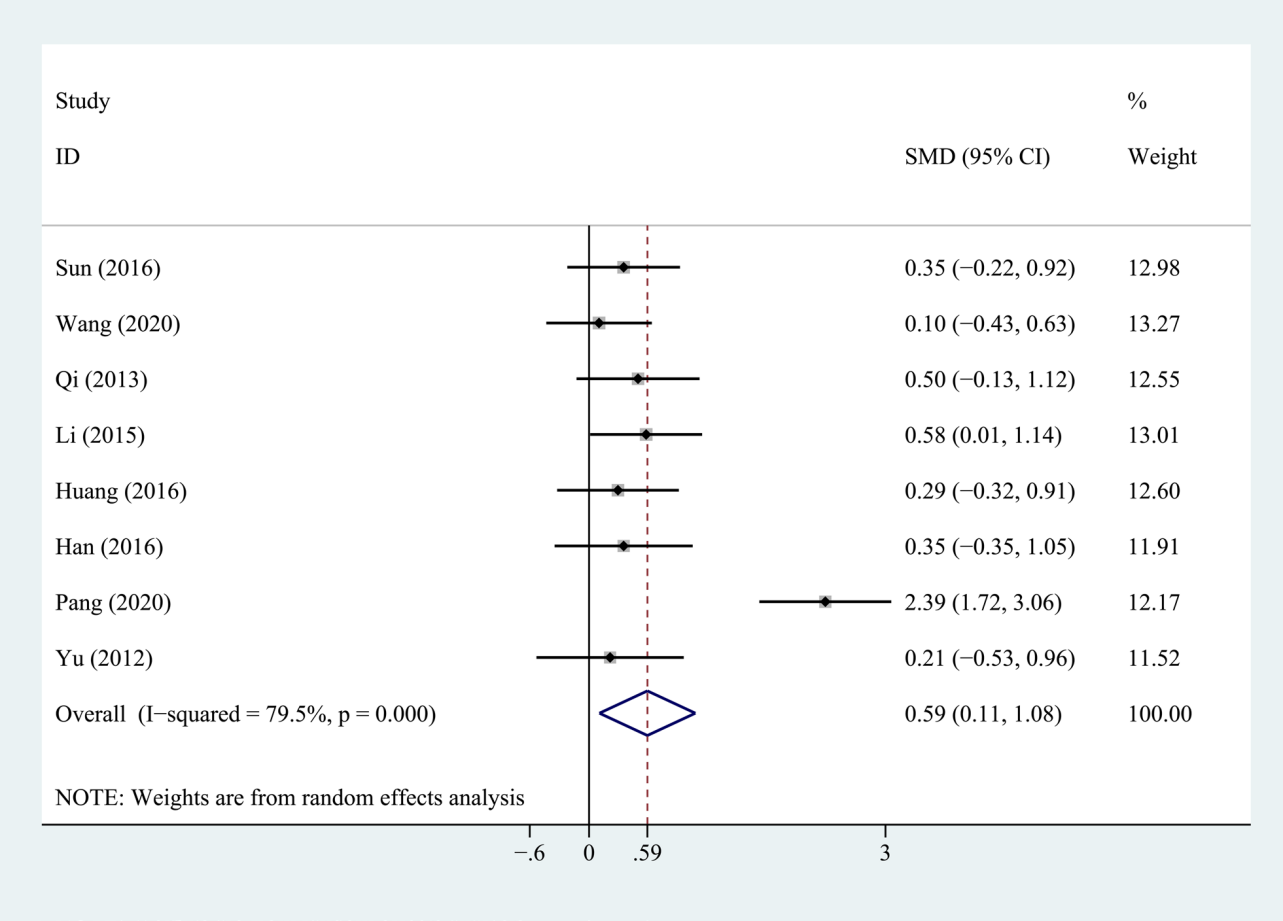
Forest plot of JOA score between PEEK and titanium rod

Eight studies compared the JOA scores between the PEEK rod group and the titanium rod group, as shown in Fig. 4. The heterogeneity test showed that there was significant heterogeneity between studies (*P* = 0.000, *I*^2^ = 79.5%), so a random-effects model was used for analysis. The combined results showed that the difference between the two groups was statistically significant (*P* = 0.017). The PEEK rod group was superior to the titanium rod group.

#### VAS score for LBP

**Fig. 5:**
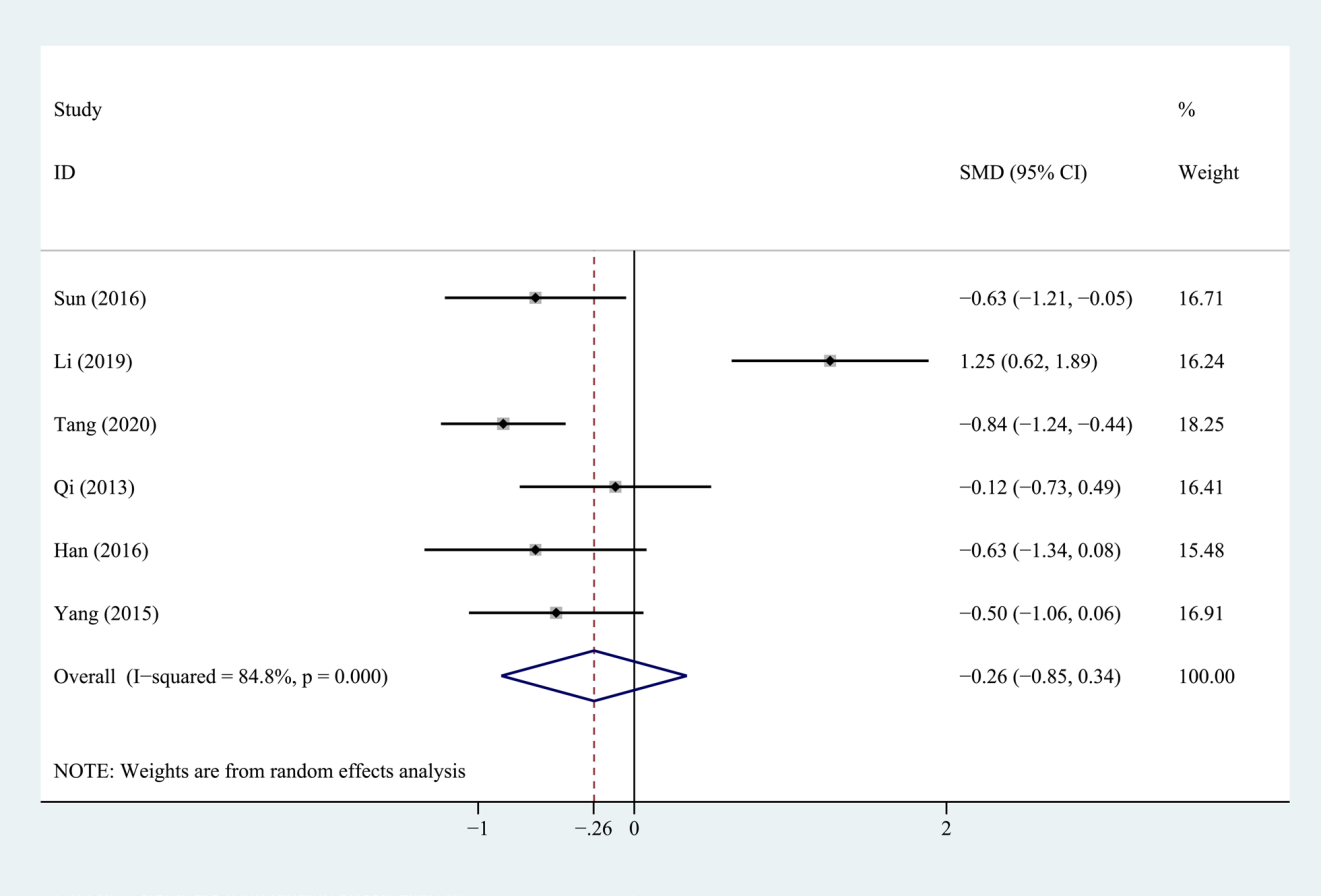
Forest plot of VAS score for LBP between PEEK and titanium rod

Six studies compared the VAS scores for LBP between the PEEK rod group and the titanium rod group, as shown in Fig. 5. The heterogeneity test showed that the heterogeneity between studies was significant (*P* = 0.000, *I*^2^ = 84.8%), so a random-effects model was used for analysis. The combined results showed that the difference between the two groups was not statistically significant (*P* = 0.396).

#### VAS score for lower limb pain

**Fig. 6:**
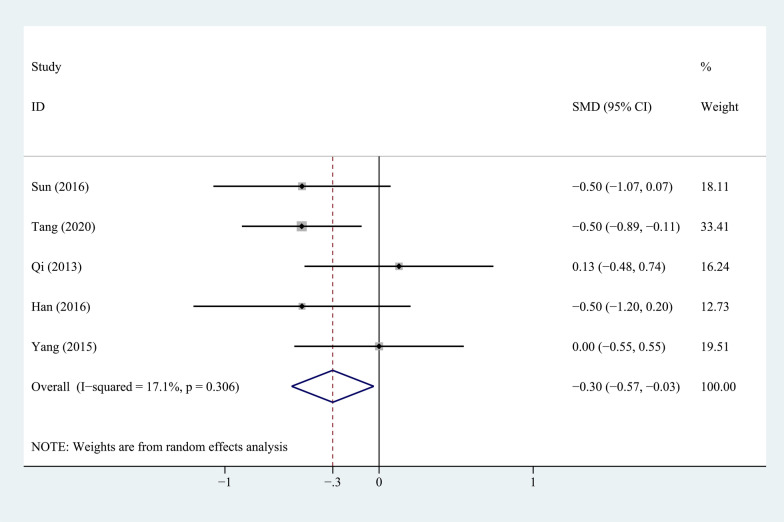
Forest plot of VAS score for lower limb pain between PEEK and titanium rod

Five studies compared the postoperative lower limb VAS scores between the PEEK rod group and the titanium rod group, as shown in Fig. 6. The heterogeneity test showed that the heterogeneity between studies was not significant (*P* = 0.306, *I*^2^ = 17.1%), so a fixed-effects model was used for analysis. The combined results showed that the difference between the two groups was statistically significant (*P* = 0.027). The PEEK rod group was better than the titanium rod group.

#### Fusion rate of bone graft at week 12

**Fig. 7:**
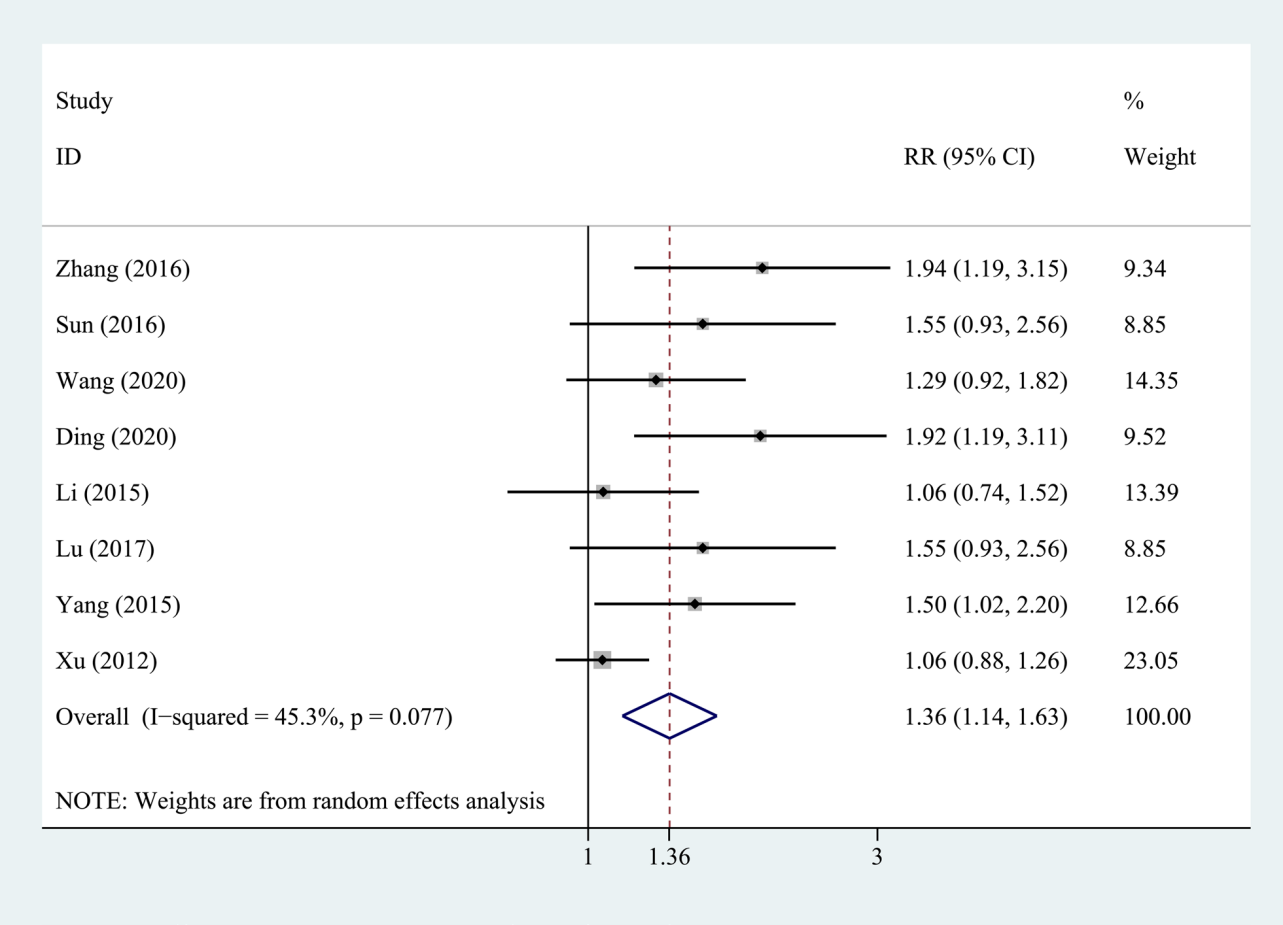
Forest plot of the fusion rate of bone graft at week 12 between PEEK and titanium rod

Eight studies compared the bone graft fusion rate at week 12 between PEEK rods and titanium rods, as shown in Fig. 7. The heterogeneity test showed that there was no significant heterogeneity between studies (*P* = 0.077, *I*^2^ = 45.3%), so a fixed-effects model was used for analysis. The combined results showed that the difference between the two groups was statistically significant (*P* = 0.001). The PEEK rod group was better than the titanium rod group.

#### Fusion rate of bone graft at last follow-up

**Fig. 8:**
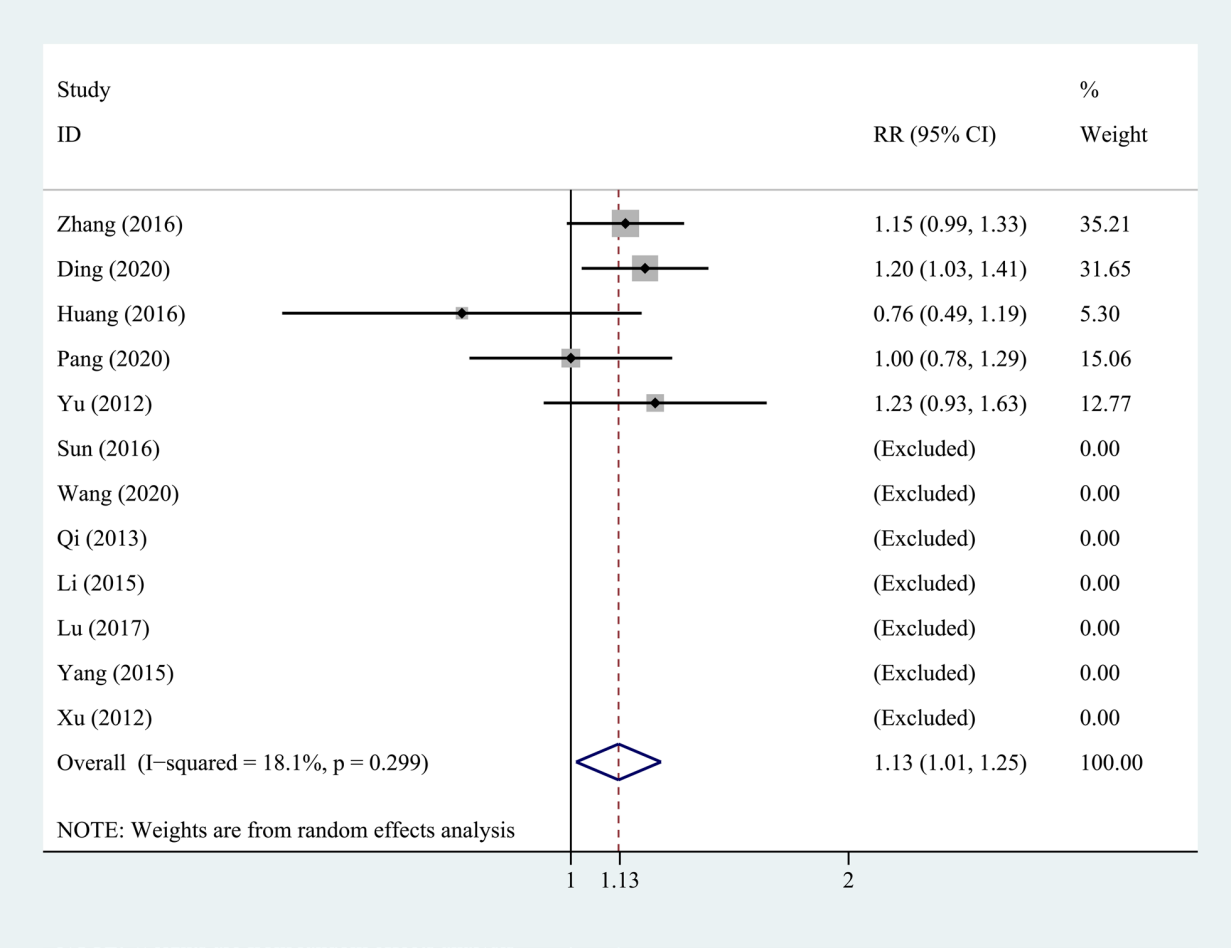
Forest plot of the fusion rate of bone graft at last follow-up between PEEK and titanium rod

Twelve studies compared the fusion rate of bone graft between PEEK rods and titanium rods at last follow-up, as shown in Fig. 8. The heterogeneity test showed that there was no significant heterogeneity between studies (*P* = 0.299, *I*^2^ = 18.1%), so a fixed-effects model was used for analysis. The combined results showed that the difference between the two groups was statistically significant (*P* = 0.028). The PEEK rod group was better than the titanium rod group.

### Sensitivity analysis

**Fig. 9:**
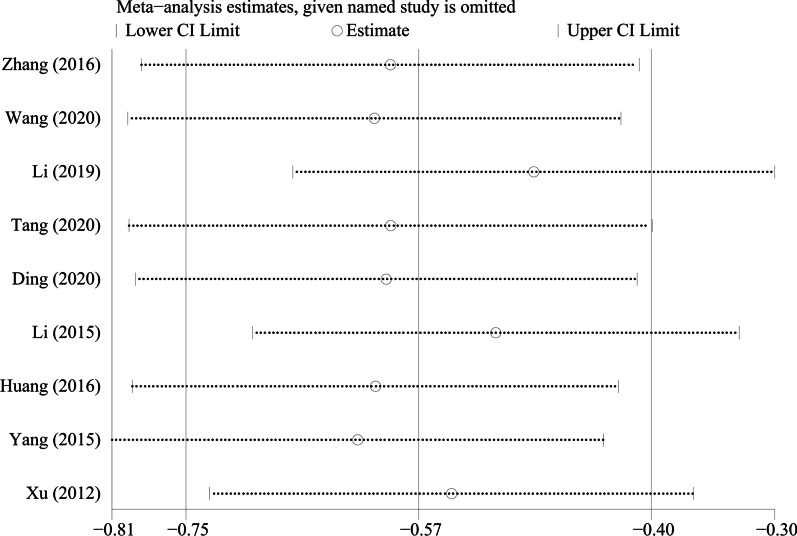
Sensitivity analysis of ODI

**Fig. 10:**
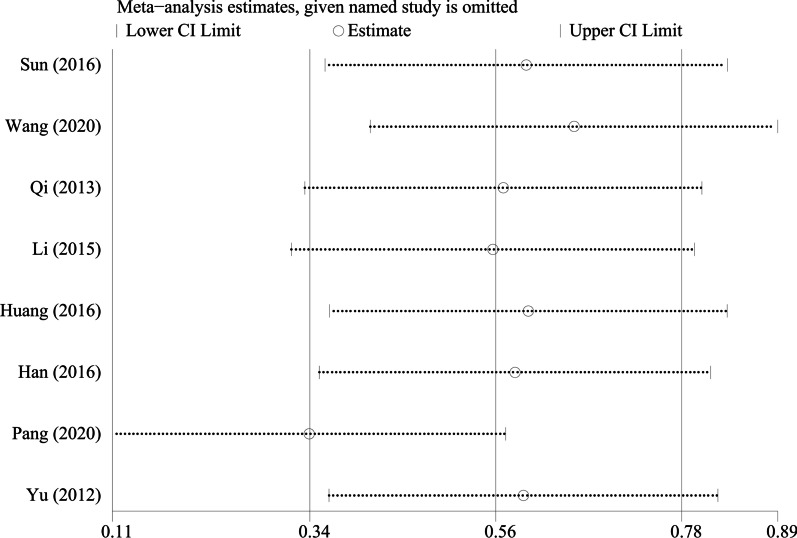
Sensitivity analysis of JOA score

**Fig. 11:**
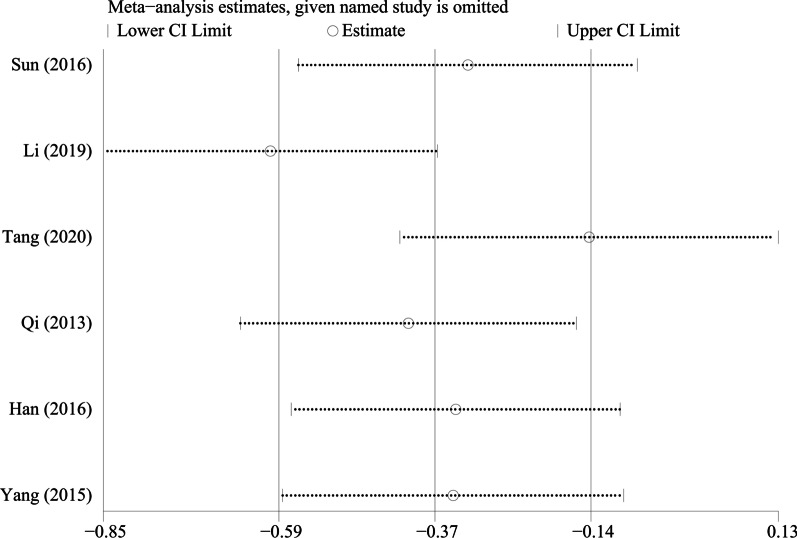
Sensitivity analysis of VAS score for LBP

**Fig. 12:**
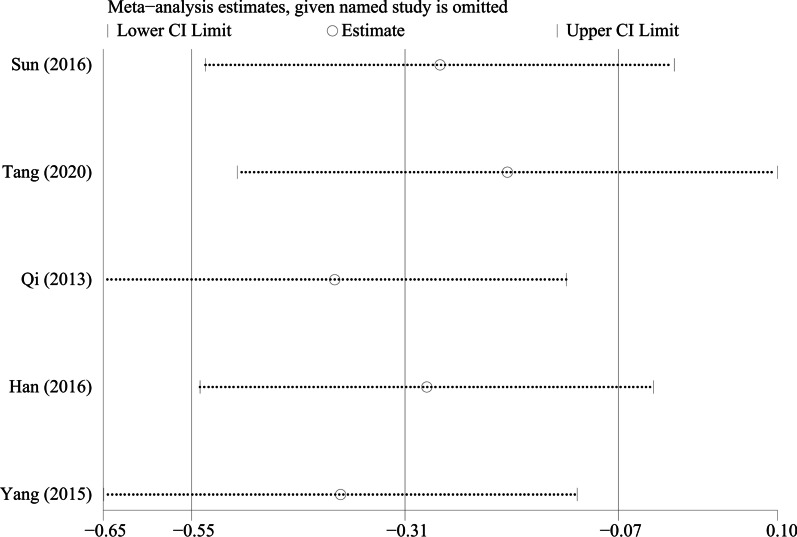
Sensitivity analysis of VAS score for lower limb pain

**Fig. 13:**
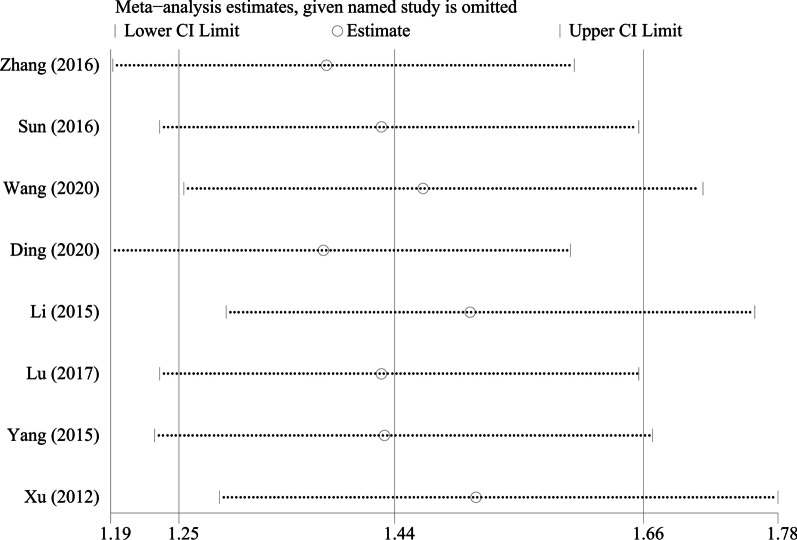
Sensitivity analysis of the fusion rate of bone graft at week 12

**Fig. 14:**
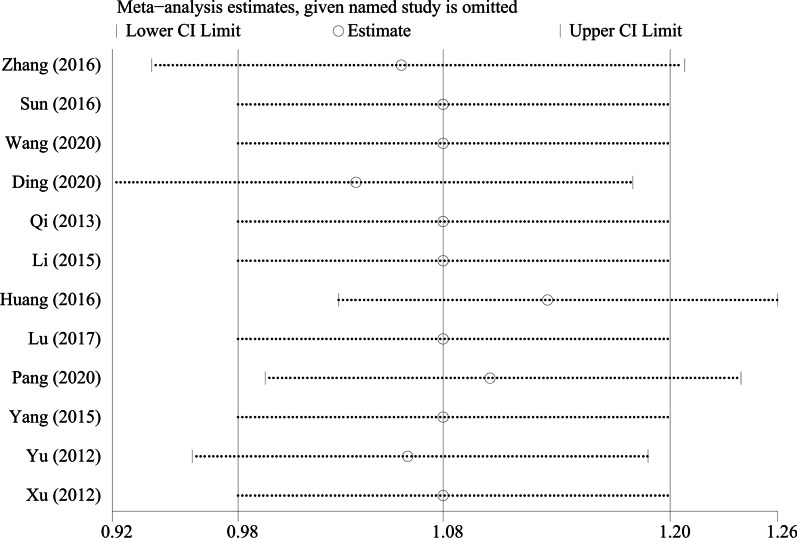
Sensitivity analysis of the fusion rate of bone graft at last follow-up

By excluding the studies one by one, we conducted a sensitivity analysis on all the outcome indicators, and drew sensitivity analysis graphs. It can be seen from the graphs that when the studies such as Pang et al. [26], Li et al. [18], and Tang et al. [19] are excluded, the combined results changed significantly, suggesting that the three studies may be a major source of heterogeneity, as shown in Figs. 9, 10, 11, 12, 13 and 14.

### Publication bias

**Fig. 15:**
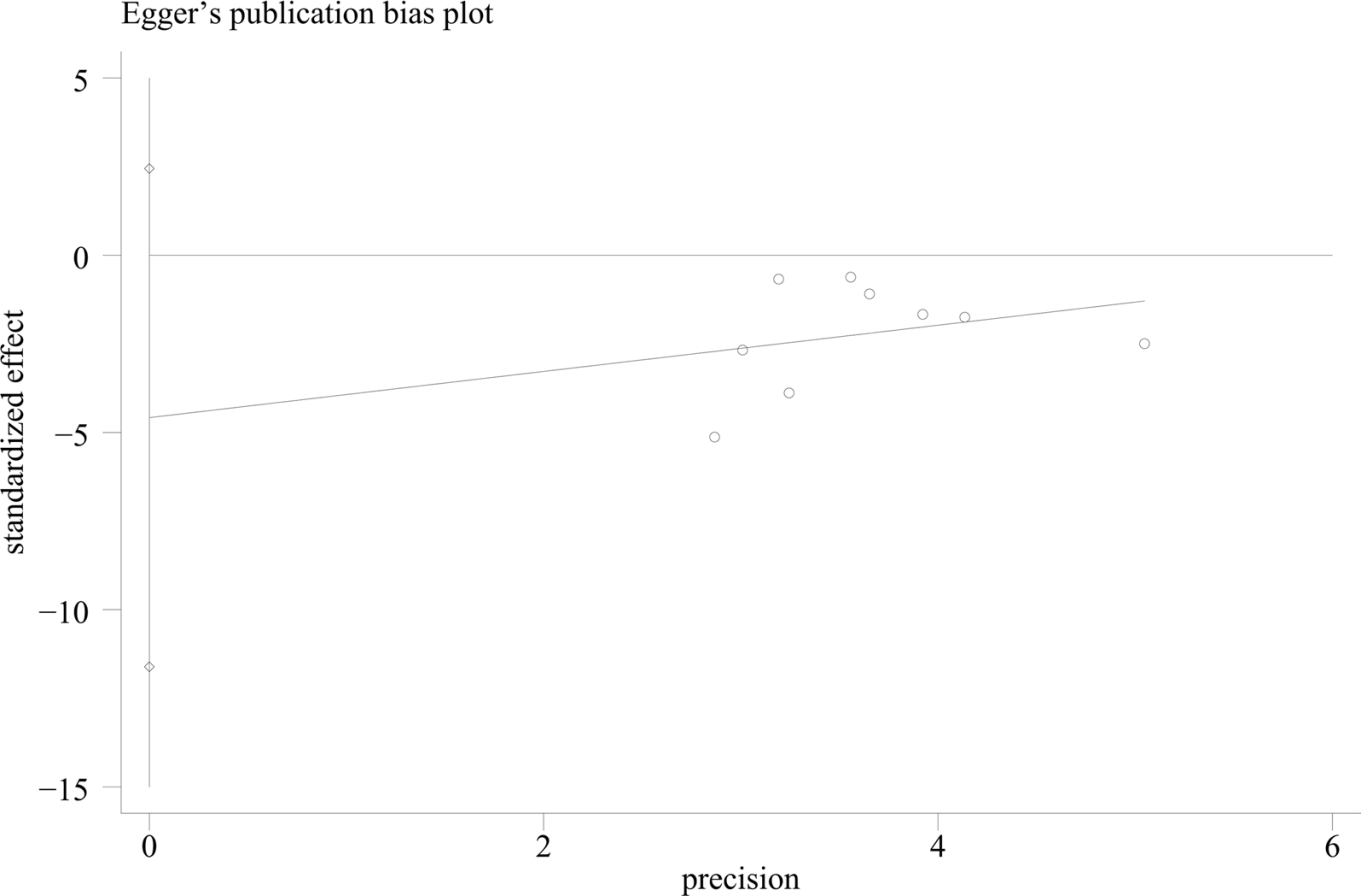
Publication bias of ODI

**Fig. 16:**
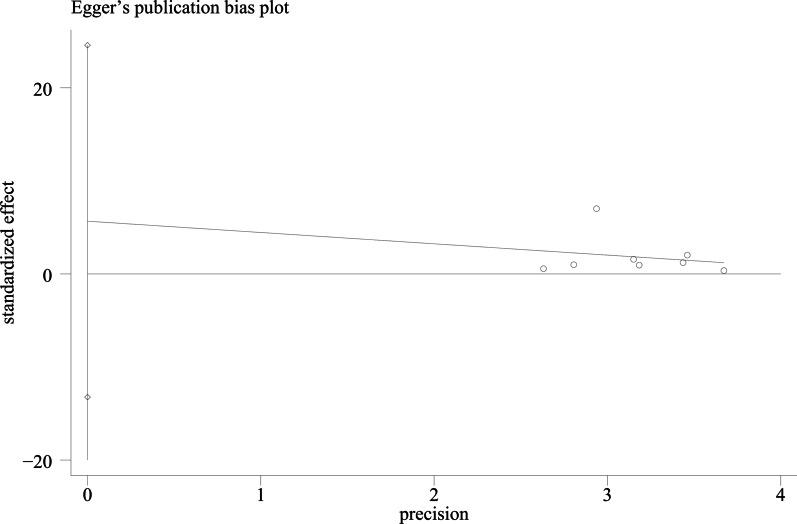
Publication bias of JOA score

**Fig. 17:**
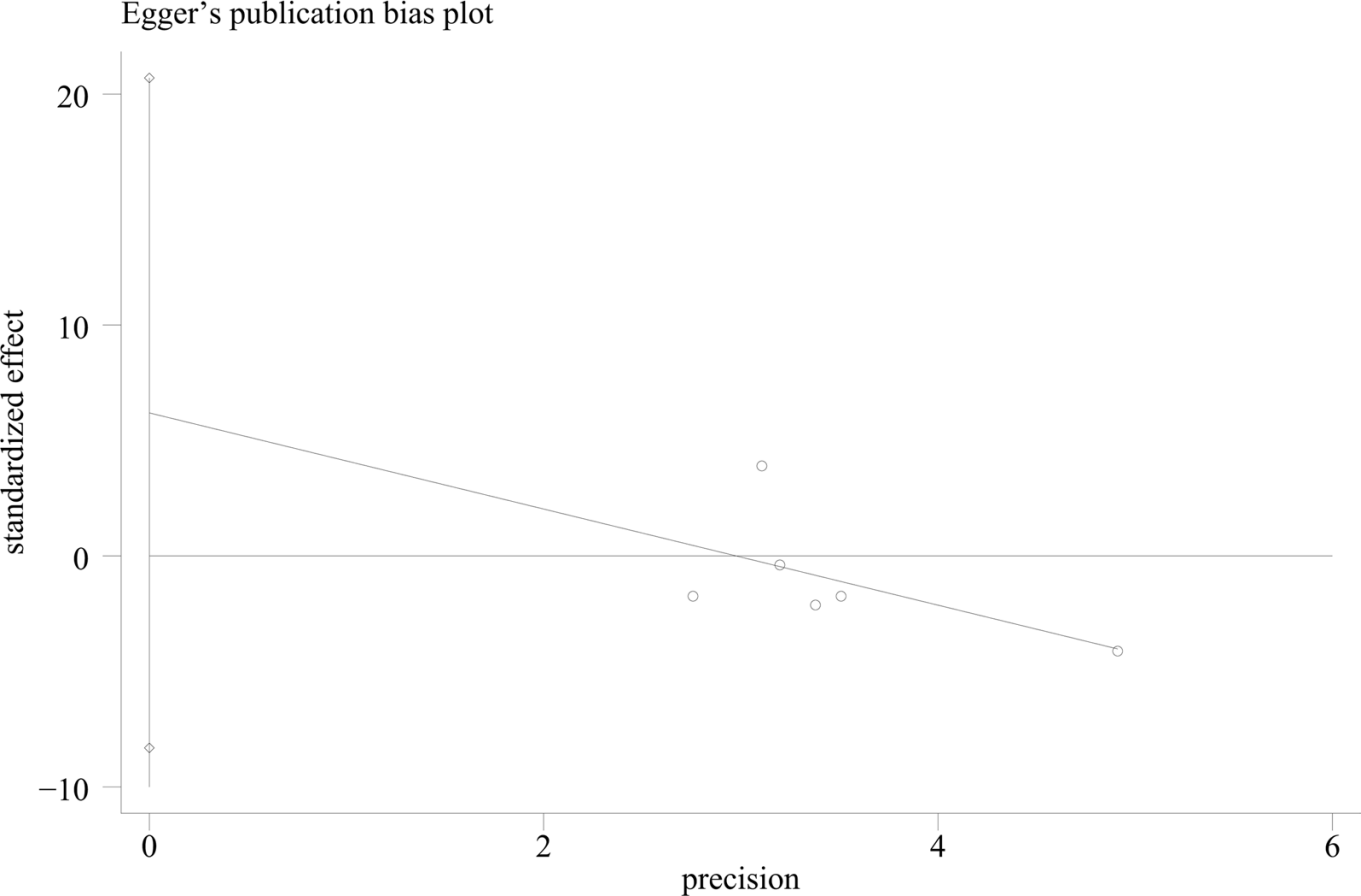
Publication bias of VAS score for LBP

**Fig. 18:**
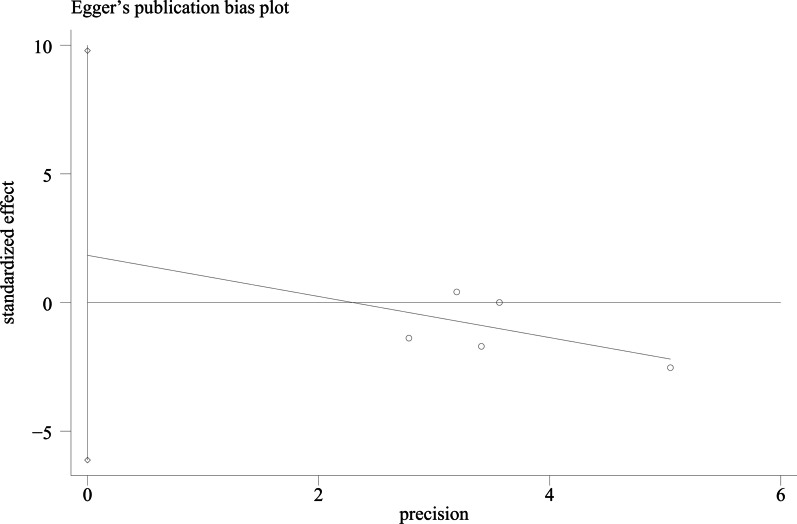
Publication bias of VAS score for lower limb pain

**Fig. 19:**
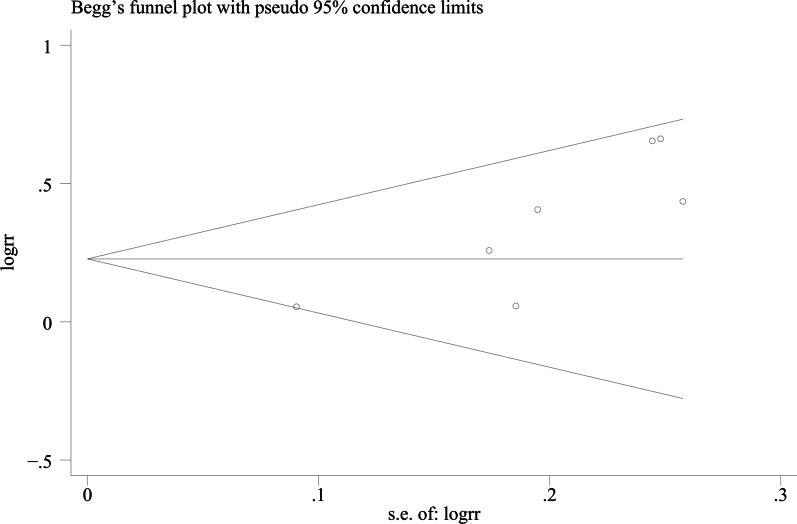
Publication bias of the fusion rate of bone graft at week 12

**Fig. 20:**
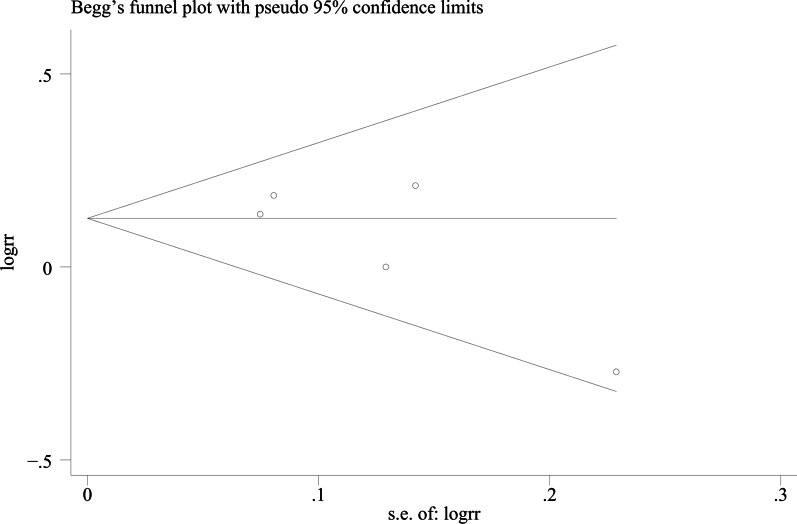
Publication bias of the fusion rate of bone graft at last follow-up

We used Egger's method and Begg's method to detect publication bias. The test results showed that ODI (*P* = 0.167), JOA score (*P* = 0.491), VAS score for LBP (*P* = 0.301), VAS score for lower limb pain (*P* = 0.516), fusion rate of bone graft at week 12 (*P* = 0.138), fusion rate of bone graft at last follow -up (*P* = 0.426) had no publication bias (*P* > 0.05), as shown in Figs. 15, 16, 17, 18, 19 and 20. It indicates that there is no selective reporting and publication of positive results in the included studies.

## Discussion

In this study, we identified 8 prospective studies and 7 retrospective studies including 779 patients to evaluate the effect of PEEK rods and titanium rods in lumbar fusion surgery. The results of the meta-analysis showed that there was no statistical difference between the two groups in the VAS score of LBP. There were statistical differences between the two groups in terms of ODI, JOA score, VAS score of lower limb pain, bone graft fusion rate at 12 weeks, and bone graft fusion rate at the last follow-up. In these aspects, the PEEK rod group was better than the titanium rod group.

Compared with the JOA score, in addition to the basic upper and lower extremity function evaluation, ODI also adds the evaluation of the patient's social life, sex, sleep, travel, etc., and the evaluation of the living ability is more detailed and comprehensive. Compared with rigid fixation with titanium rods, semi-rigid fixation with PEEK rods allows a greater ROM of the lumbar spine. Finite element analysis [30] showed that the ROM values of PEEK rods in axial rotation, lateral bending and buckling were increased by 3.7, 7.2 and 2.15 times compared with titanium rods, respectively. This helps to reduce the stiffness and restraint of postoperative patients during daily activities such as putting on and taking off clothes, standing, walking, washing, etc., which is beneficial to improve the comfort of patients, and also reduces the difficulty of postoperative rehabilitation training.

In terms of bone graft fusion rate, our study showed that the bone graft fusion rate of PEEK rod group was higher than that of titanium rod group at the 12th week after operation or at the last follow-up, indicating that semi-rigid fixation may be more beneficial to bone graft fusion. This is contrary to our intuition, because in our perception, the strong fixation of titanium rods seems to represent a higher implant fusion rate. Since the elastic modulus (3.2 GPa) of PEEK material is between cortical bone and cancellous bone and significantly lower than that of titanium (114 GPa) [31], it is more mechanically compliant to the spine. It can better mimic the load distribution in the physiological environment of the lumbar spine. Under physiological loading conditions, the PEEK rod structure can increase the front column load by about 75% compared with the titanium rod [32]. Increased loading of the anterior column creates greater stress on the bone graft area and also allows for greater contact between the endplate and the bone graft. Coupled with the micro-motion generated by the elastic rod in the bone graft area, it may be the reason why PEEK rods promote the fusion of the bone graft [33–35].

In terms of VAS for LBP, there was no statistical difference between the two groups, indicating that semi-rigid immobilization may not bring more pain in the surgical area while increasing the ROM of the lumbar spine after surgery. The PEEK rod group appeared to be superior to the titanium rod group in VAS for lower limb pain, which was not explained in all included studies. Qi et al. [21] believed that there is no difference in the degree of intervertebral space height loss between PEEK rods and titanium rods, and both can meet the requirements of intervertebral space height. Biswas et al. [30] believed that for pedicle screw fixation, no matter what kind of rod was used, the height of the intervertebral foramen did not change significantly, and it would not interfere with the nerve root. We believe that this may be related to the greater lumbar ROM and better spinal compliance afforded by the PEEK rods. We believe that a more comfortable postoperative feeling will encourage patients to perform more rehabilitation and daily activities, which will help promote the reduction of nerve edema and inflammation, thus reducing postoperative lower extremity pain.

Reducing the incidence of ASD is the main purpose for which PEEK rods were created. This mechanism can be broadly explained in two ways: one is the increase of fixed segment activity, which reduces the compensatory overactivity of adjacent segments; the other is the load distribution pattern that is closer to the physiological state [1–3]. Unfortunately, none of the studies we included reported the occurrence of ASD, which we believe may be related to the short follow-up period. Of the 15 included studies, only Sun et al. [16] had a follow-up time of 4 years, and the follow-up time of the rest of the studies was within 3 years. In our opinion, this does not seem to be enough to observe the occurrence of ASD.

In terms of screw stability, according to the study of Gornet et al. [32], the PEEK rods can reduce the load of the bone-screw interface by about 25% while increasing the load of the anterior column, reducing the stress shielding, thereby reducing the risk of screw loosening, especially in osteoporotic bone [36]. Wu et al. [37] observed denser and thicker trabecular bone around the screw in the PEEK rod group in the sheep cervical fusion model, and believed that the PEEK rod could produce better biomechanical distribution and promote the growth of trabecular bone around the screw. In terms of strength and durability, although it has been suggested [38] that the ratio of peak stress to material yield stress of PEEK rods in physiological state is higher than that of titanium rods, which will increase the risk of rod fracture. But more studies hold the exact opposite view. Study of Agarwal et al. [39]showed that the motion data of PEEK rods before and after fatigue were not significantly different from those of titanium rods with significantly higher motion after fatigue than before. Moreover, studies [40]have shown that PEEK rods can withstand static and fatigue angular displacements that exceed five times that of the cadaveric test recommendations without fracture, torsion, yield and plastic deformation in static and dynamic compression bending tests and torsional tests. Wang et al. [35] tested PEEK rods of various diameters in a canine model, and the results showed that even the thinnest PEEK rod (2.0 mm, 197N) had a significantly higher yield load than the lumbar spine stress (17.5N). In addition to the above studies, there are several biomechanical studies that suggest that the stability provided by PEEK rods in posterior lumbar fusion is not significantly different from that of titanium rods [30, 32, 41]. Moreover, PEEK materials can also customize stiffness through carbon fiber reinforcements, which may have advantages over conventional materials [42].

In terms of adverse events, Hirt et al. [43] reviewed 462 patients and showed that the PEEK rod group was less likely to be readmitted due to adverse events. Ross et al. [44] reviewed 108 patients and also showed a lower complication rate. None of the 15 studies we included reported any adverse events such as loosening of internal fixation, rupture, nerve injury, and infection. However, despite these advantages, PEEK is not a perfect material for internal fixation. First, its radiolucency, while reducing artefacts in CT scans and making radiological follow-up easier, may fail to detect damage in the event of a breakage. Another disadvantage is cost, PEEK material is more expensive than titanium. Therefore, use without indications may result in a substantial increase in the cost of surgery [21, 34]. It has also been suggested [36] that although PEEK rods can promote bone graft fusion, there is a risk of cage subsidence and injury. In addition, PEEK rods have no spinal correction effect [21].

### Limitation

Our meta-analysis has the following limitations. First, the number of studies that met the criteria was small and they were all limited to one country, and their quality was not high enough. Secondly, there is a lack of indicators to evaluate subjective feelings such as patient comfort and satisfaction. Moreover, the included studies were heterogeneous, which may be related to the large differences in surgical operation habits, surgical segments, and follow-up time of different investigators. Finally, the follow-up time of the included studies was not long enough, the long-term efficacy, incidence of adverse events, and degeneration of adjacent segments between the two groups remain to be tested and compared.

## Conclusion

In conclusion, both PEEK rods and titanium rods can provide reliable fixation in lumbar fusion surgery. PEEK rods may be better than titanium rods in improving postoperative dysfunction, reducing lower limb pain, and improving bone graft fusion rate. However, given the limitations of this study, the applicability of these conclusions remains to be further investigated. Our work contributes to a more rational view of PEEK materials and the semi-rigid fixation represented by PEEK rods in lumbar fusion surgery.

## Data Availability

All data generated or analysed during this study are included in this published article and its supplementary information file.

## Abbreviations

PEEK: Polyetheretherketone
MD: Mean differences
OR: Odds ratios
CI: Confidence intervals
JOA: Japanese Orthopaedic Association
VAS: Visual Analog Scale
LBP: Low back pain
ODI: Oswestry Disability Index
ASD: Adjacent segment degeneration
PRISMA: Preferred Reporting Items for Systematic Reviews and Meta-Analyses
CNKI: China National Knowledge Infrastructure
ROM: Range of motion
DH: Disc space height
